# Investigation of the relationship between contact lens design parameters and refractive changes in Ortho-K

**DOI:** 10.1016/j.heliyon.2022.e11699

**Published:** 2022-11-19

**Authors:** Lo-Yu Wu, Louise Pellegrino Gomes Esporcatte, Wen-Kai Li, Wen-Pin Lin, Richard Wu, Lynn White, Marcella Q. Salomão, Bernardo T. Lopes, Renato Ambrósio, Ahmed Abass

**Affiliations:** a Department of Power Mechanical Engineering, Nation Tsing Hua University, Hsinchu, Taiwan; b Research and Development Center, Brighten Optix Corporation, Taipei, Taiwan; c Rio de Janeiro Corneal Tomography and Biomechanics Study Group, Rio de Janeiro, Brazil; d Instituto de Olhos Renato Ambrósio, Rio de Janeiro, Brazil; e Department of Ophthalmology, Federal University of São Paulo, São Paulo, Brazil; f Department of Optometry, University of Kang Ning, Taipei, Taiwan; g College of Optometry, Pacific University, Forest Grove, OR, USA; h Research and Development Department, LWVision, Leicester, UK; i Brazilian Study Group of Artificial Intelligence and Corneal Analysis – BrAIN, Rio de Janeiro & Maceió, Brazil; j Instituto Benjamin Constant, Rio de Janeiro, Brazil; k Department of Civil Engineering and Industrial Design, School of Engineering, University of Liverpool, Liverpool, UK; l Department of Ophthalmology, Federal University the State of Rio de Janeiro, Rio de Janeiro, Brazil; m Department of Mechanical, Materials and Aerospace Engineering, School of Engineering, University of Liverpool, Liverpool, UK; n Department of Production Engineering and Mechanical Design, Faculty of Engineering, Port Said University, Port Said, Egypt

**Keywords:** Eye, Cornea, Ortho-K, Contact lenses, Target power change

## Abstract

**Purpose:**

To investigate the relationship between Ortho-K contact lens design parameters and refractive power change of the eye through a parametric mathematical representation.

**Methods:**

The current study utilises fully anonymized records of 249 eyes, 132 right eyes, and 117 left eyes from subjects aged 14.1 ± 4.0 years on average (range 9–38 years) which were selected for secondary analysis processing. The data were split into 3 groups (G1 up to 35 days wear, from 10 to 35 days, G2 up to 99 days wear, more than 35–99 days & G3 more than 100 days wear) according to the length of time, in days, that the lenses were worn. Corneal shape was measured before and after contact lens wear using the Medmont E300 topographer, from which height and distance files were read by a custom-built MATLAB code to construct the corneal anterior surface independently. Changes in refractive power pre and post-Ortho-K wear were determined using constructed tangential refractive power maps from which both centrally flattened and annular steepened zones were automatically bounded, hence used to determine the refractive power change.

**Results:**

On average, flat Sim-K and steep Sim-K were reduced after Ortho-K lens wear by 1.6 ± 1.3 D and 1.3 ± 1.4 D respectively. The radius of the base curve was correlated with the mean central flattened zone power change strongly in G1 (R = 0.7, p < 0.001) and moderately in G2 (R = 0.4) and G3 (R = 0.4, p < 0.001). Hence, a strong correlation with the base curve was recorded in group G1 and moderate in G2 and G3. The reverse curve was very strongly correlated to the mean central flattened zone power change in G1 (R = 0.8, p < 0.001) and strongly correlated with G2 (R = 0.6, p < 0.001) and G3 (R = 0.7, p < 0.001). The reverse curve was also strongly correlated with the mean annular steepened zone power change among all groups G1, G2, and G3 (R = 0.7, R = 0.6 and R = 0.6) respectively (p < 0.001).

**Conclusions:**

Although the central corneal refractive power change was strongly correlated to the Ortho-K lens base curve, it characterized only 50% of the target power change. However, the annular steepened zone refractive power change appears to be a clearer predictor of target power change, as there appears to be a one-to-one inverse relationship with the target refractive power correction. Differences between these results and the literature may be a result of the topography software smoothing effect.

## 1 Introduction

Myopia is a common eye condition that is likely to show a significant increase in global prevalence by 2050 as estimated by Holden et al. (2016). High myopia presents significant visual challenges in the form of cataracts, glaucoma, retinal detachment, and myopic macular degeneration, all of which can cause substantial vision loss. There is consequently a strong interest in attempting to slow the progress of myopia but although several different methodologies have been developed, none have yet proven to be more than around 50% successful in slowing axial length growth. One of the more widely used methodologies is prescribing Orthokeratology (Ortho-K) contact lenses, which were initially designed to reshape the anterior cornea to reduce refractive error (reversibly and temporarily) after wearing them overnight.

Ortho-K lenses are typically designed so that the back surface consists of four back surface curves; the back central optic zone (BOZ), a reverse curve, and two alignment zones. The BOZ flattens the central cornea, effectively reducing its dioptric power, and the related area on the cornea has been defined as the Treatment Zone (TZ) (Faria-Ribeiro et al., 2016; Carracedo et al., 2019). The reverse curve creates a space between the posterior lens surface and the anterior corneal surface, allowing it to fill with tears. This then forms a pressure gradient, with positive pressure centrally and negative pressure peripherally, resulting in epithelial migration from the central cornea to an annular, peripheral steepening zone (PSZ) corresponding to the reverse curvature position, thus initiating peripheral corneal thickening (Swarbrick 2006; Zhang et al., 2020; Vincent et al., 2021). The refractive effect of this PSZ may influence myopia control (Charman, 2006; Gifford et al., 2020).

Ortho-K treatment is monitored using topography machines, such as the Medmont E300 instrument. The TZ and the PSZ can be determined using tangential, axial and refractive differential maps and then comparing the treated corneal area to the baseline measurements. Understanding the relationship between these zones, target power and the central corneal power change is essential to enable modification of fitting characteristics to optimise the performance of the treatment, whether this is refractive error reduction or myopia control.

For example, some studies have investigated the relationship between smaller TZ and greater peripheral power change (Pauné et al., 2021; Zhang et al., 2022), suggesting a smaller BOZ leads to a greater peripheral corneal power change. Others have examined the effect on myopia control outcomes of decentration of the TZ (Chen et al., 2018; Gu et al., 2019; Wang and Yang, 2019). However, drawing clinical conclusions about such changes requires a reliable and repeatable methodology. A recent study compared manual and software-based measurement systems to measure TZ and PSZ and found that they were significantly different, although clinically acceptable (Guo et al., 2021). However, the differences in refractive power measurements were clinically significantly different, which the authors attributed to the effects of the topographer software algorithms.

This study examines the potential relationships between TZ diameter, the reverse curve width (RCW), target power and central flattening, and annular peripheral power changes using a retrospective study of patient records. The methodology for measuring changes to the cornea is software-based rather than manual and utilises extraction of raw height data from the topographer to avoid any effects from inbuilt software algorithms.

## 2 Methods

### 2.1 Contact lens design

All contact lenses used in the study were of the same Hiline Ortho-K design manufactured from Boston XO (hexafocon A) with a violet handling tint and oxygen permeability (DK) of 100 × 10^−11^ (cm^3^O_2_/cm^2^s). All contact lenses were manufactured by Brighten Optix Corporation (Taipei, Taiwan) and the design comprises 4 back curves: a central base curve (Back Optic Zone Diameter 6.00mm), a reverse curve, and 2 consecutive alignment curves with a 0.22 mm central thickness and diameters ranging from 10.00 mm to 10.80 mm.

### 2.2 Subject data collection and processing

This retrospective study utilised fully anonymised records of 249 eyes fitted with Hiline Ortho-K lenses. One eye per patient was chosen randomly, resulting in a total of 132 right eyes and 117 left eyes. The average age of the subjects was 14.1 ± 4.0 years (range 9–38 years) with spherical refraction of up to -10.00 DS and the data was used solely for secondary analyses processing. Informed consent was obtained from all participants in the study which was conducted following the standards set in the Declaration of Helsinki and approved by the ethical committee board of the Federal University of São Paulo (UNIFESP/SP 2020 # 4.050.934). Only participants with no history of ocular disease, binocular anomaly, trauma, or ocular surgery were included. All participants were instructed to wear their Ortho-K lenses every night and only fully compliant participants were included. Those subjects who suspended wear for a few days as a result of corneal abrasion, or any other reason, were excluded from the analyses. Early morning measurements were exclusively included to ensure that the topography maps included in the analyses were representative and to minimize any discrepancy as a result of the variation between morning and afternoon measurements. This was achieved automatically using the MATLAB software's “textscan” function to read the “EntryDate” data element from each Medmont E300 ∗.mxf file which supplied both the date and the entry time of the topography measurement, allowing the date to be filtered to only select readings taken before 10 am on the follow-up date.

As this is a retrospective study of an active clinic, the subjects are representative of new and established wearers, the data were organized into groups of subjects who experienced similar wear time of the Ortho-K contact lenses. Group (G1) represents subjects with a wear time of 10–35 consecutive days (from 10 to 35 days, n = 32). The second group (G2) represents subjects with a wear time of 99 consecutive days (more than 35–99 days, n = 79) and finally, a third group (G3) with at least 100 consecutive days of wear and over (more than 99 days, n = 138).

Table 1. The groups were analysed to ensure they were similar in terms of demographics, age, central Sim-K readings and corneal asphericity.

**Table 1:** The three groups of participants selected for the study based on the time of wearing Ortho-K lenses.

Groups	Time of wear (days)	Number of eyes	Age in years (m ± std)	Pre-Simulated keratometry (Sim-K) (D)		Post-Simulated keratometry (Sim-K) (D)		Pre-Wear Asphericity		Post-Wear Asphericity		Eye Side	
				Flat (m ± std)	Steep (m ± std)	Flat (m ± std)	Steep (m ± std)	Flat (m ± std)	Steep (m ± std)	Flat (m ± std)	Steep (m ± std)	Right	Left
The first group (G1)	Up to 35 days of wear 10 ≤ time ≤ 35	32	15.6 ± 4.3	42.43 ± 1.09	43.74 ± 1.20	41.20 ± 0.99	42.70 ± 0.99	0.66 ± 0.12	0.42 ± 0.13	0.34 ± 0.17	0.35 ± 0.13	15	17
The second group (G2)	Up to 99 days of wear 35 < time ≤ 99	79	14.9 ± 5.1	42.69 ± 1.40	44.06 ± 1.34	41.11 ± 1.47	42.76 ± 1.63	0.65 ± 0.09	0.36 ± 0.15	0.34 ± 0.15	0.41 ± 0.15	39	40
The third group (G3)	More than 99 days of wear time ≥ 100	138	13.4 ± 3.0	42.62 ± 1.31	43.95 ± 1.38	41.01 ± 1.32	42.68 ± 1.51	0.67 ± 0.10	0.42 ± 0.19	0.34 ± 0.14	0.45 ± 0.18	78	60
All g roups	10 ≤ time ≥ 100	249	14.10 ± 4.0	42.62 ± 1.31	43.96 ± 1.34	41.07 ± 1.33	42.71 ± 1.49	0.66 ± 0.10	0.40 ± 0.18	0.34 ± 0.14	0.43 ± 0.17	132	117

All patients were measured using the E300 Medmont topographer instrument and height files “∗.hgt” and distance files “∗.dst” were digitally scanned and extracted by a custom-built MATLAB code. From this, a grid of 300 angular and 333 radial positions was used to construct the corneal anterior surface. Medmont E300 “∗.mxf” files were also digitally scanned via the code to extract simulated Keratometry values FlatK, SteepK, FlatAngle and SteepAngle as well as the date and time of the measurement.

### 2.3 Contact lens fitting protocol and follow-up

Initially, the refractive error of the study patients was assessed using an autorefractor followed by subjective refraction, with Best Corrected Visual Acuity (BCVA) recorded in decimal format using a Snellen illuminated chart at 6 m. The patients were fitted with ortho-K contact lenses using the standard procedure outlined by the manufacturers of the contact lenses. HVID, flattest corneal curvature (FK) and the target power (TP) for each patient were collected and a nomogram was used to select the appropriate lens design. Once the lenses were manufactured, the ECP evaluated the fit and a new lens(es) would be ordered, if appropriate. Once the fit was confirmed, the lenses were dispensed, and patients were instructed to wear them overnight for at least 8 h each night for at least a week and then to return to the clinic for follow-up and any further adjustments. The patients were then followed up one month later and then at regular intervals. The minimum wearing time included in the current study was 10 days. Once the patient was satisfied with the visual result, the final prescription was measured using the autorefractor, the result was checked subjectively and the final visual assessment was recorded without contact lens or spectacle correction, Table 2.

A hydrogen peroxide lens care regime was issued to all patients with instructions to use it to clean their lenses every day and to use Progent A disinfectant (Menicon Co., Ltd., Japan) every month to remove any excess lipids or protein build-up on the contact lens surface.

### 2.4 Tangential refractive power change

Clinically observed tangential refractive power changes were determined following the method described in (Lu et al., 2007) where the corneal power obtained via tangential curvature pre and post-Ortho-K wear illustrated the central flattened zone and the annular steepened zone. Briefly, tangential curvature maps of both pre and post-Ortho-K wear corneas were produced. Then, to allow a common coordinate among pre and post-corneal topographies, 3D triangulation-based cubic interpolation (Sandwell, 1987) was used to reconstruct the post-Ortho-K wear cornea so that it was axially aligned with the pre-Ortho-K cornea by sharing the same XY coordinates. The Medmont E300 software has signal processing elements that are unknown to users and therefore cannot be anticipated. To allow independent analysis, a custom-built MATLAB function was used to determine the tangential curvature. With cartesian coordinates covering the range -6 to 6 mm in both the X direction (nasal-temporal) and Y direction (superior-inferior), the Z-axis represents the anterior eye surface height (raw elevation). Radial direction distance from the corneal longitudinal Z-axis is represented by r (Eq. (1)) where

(1) $$r=\sqrt{X^{2}+Y^{2}}$$

and tangential radius of curvature $R_{t}$ can be expressed in terms of the first and second derivatives of the height Z relative to the radial distance by the differential equation Eq. (2).

(2) $$R_{t}=\frac{{\left(1+{\left(\frac{dZ}{dr}\right)}^{2}\right)}^{\frac{3}{2}}}{\frac{d^{2}Z}{dr^{2}}}$$

The corneal net refractive power $P_{net}$ is typically determined by using the Gaussian optics formula (Olsen, 1986; Ho et al., 2008) as

(3) $$P_{net}=\frac{n_{cornea}-n_{air}}{R_{anterior}}+\frac{n_{aqueous}-n_{cornea}}{R_{posterior}}-\frac{CCT}{n_{cornea}}\times \frac{n_{cornea}-n_{air}}{R_{anterior}}\times \frac{n_{aqueous\;}-n_{cornea}}{R_{posterior}}$$

where the refractive indices of air, $n_{air}$, cornea, $n_{cornea}$, and aqueous, $n_{aqueous}$, are set to 1.0, 1.376 and 1.336, respectively, following Gullstrand's relaxed eye model (Smit and Atchison, 1970; Vojnikovi and Tamajo, 2013). As the Medmont E300, like other Placido topographers, does not measure the corneal posterior surface, nor the central corneal thickness CCT, only anterior radii of curvature $R_{anterior}$ can be used to approximately determine the net corneal refractive power. In this case, $R_{anterior}$ was set to $R_{t}$ and Eq. (3) was reduced to Eq. (4) where $n_{cornea}$ was set to a hypothetical value of $n_{h}=1.3375$ working as a correction element compensating for the absence of the posterior corneal refractive power component. Hence, the tangential refractive power $P_{t}$ could be expressed as in Eq. (4);

(4) $$P_{t}=\frac{n_{h}-n_{air}}{R_{t}}$$

**Figure 1:**
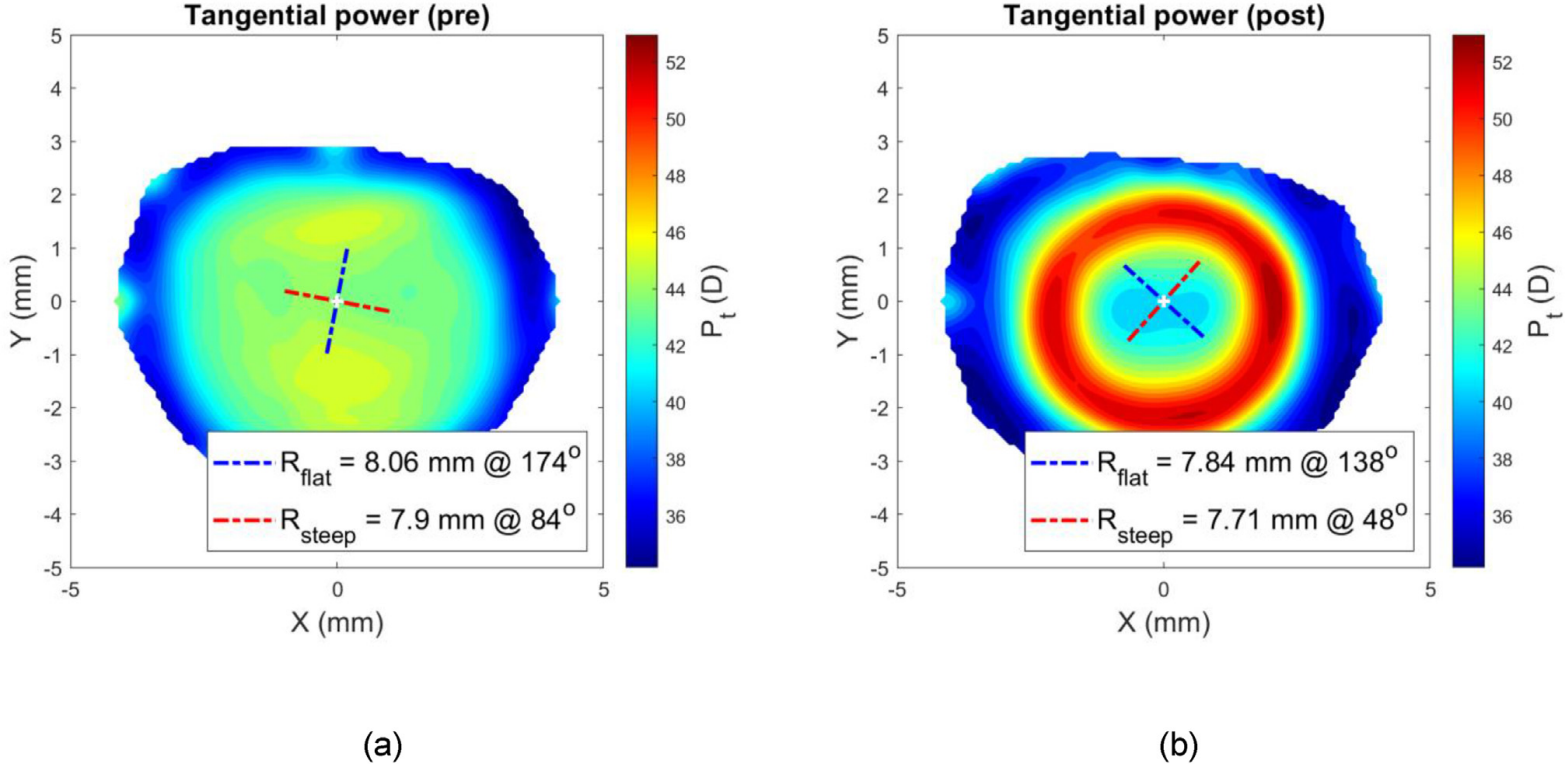
An example of a right eye of a 21-year-old male subject where the corneal pre (a) and post (b) Ortho-K wear tangential refractive power maps were determined by a custom-built MATLAB code.

At this stage, the difference between the post-Ortho-K wear corneal tangential refractive power and pre-Ortho-K wear tangential refractive power, $\Delta P_{t}$ could then be established as a map according to Eq. (5), Figure 1.

(5) $$\Delta P_{t}=P_{t_{post}}-P_{t_{pre}}\;where\;P_{t_{pre}}=\frac{n_{h}-n_{air}}{R_{t_{pre}}}\;\&\;P_{t_{post}}=\frac{n_{h}-n_{air}}{R_{t_{post}}}$$

**Figure 2:**
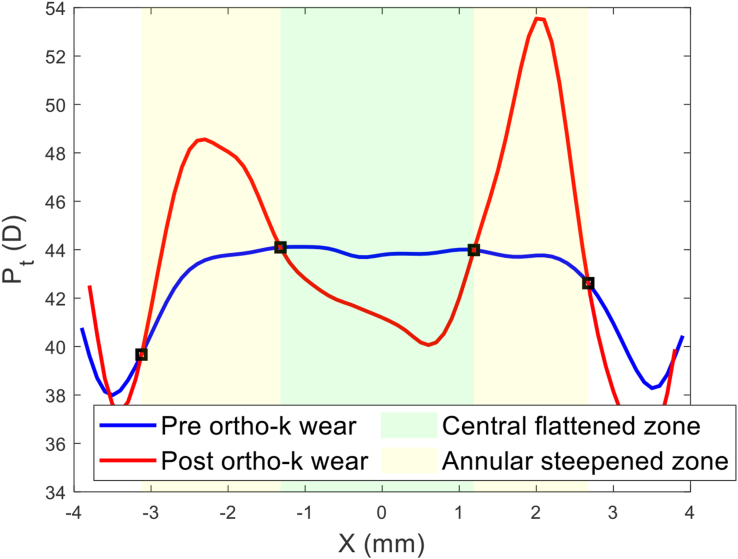
Nasal temporal section in which both central flattened zone and annular steepened zone were detected by locating the intersection between the pre and post Ortho-K tangential refractive power profiles at the nasal temporal meridian.

**Figure 3:**
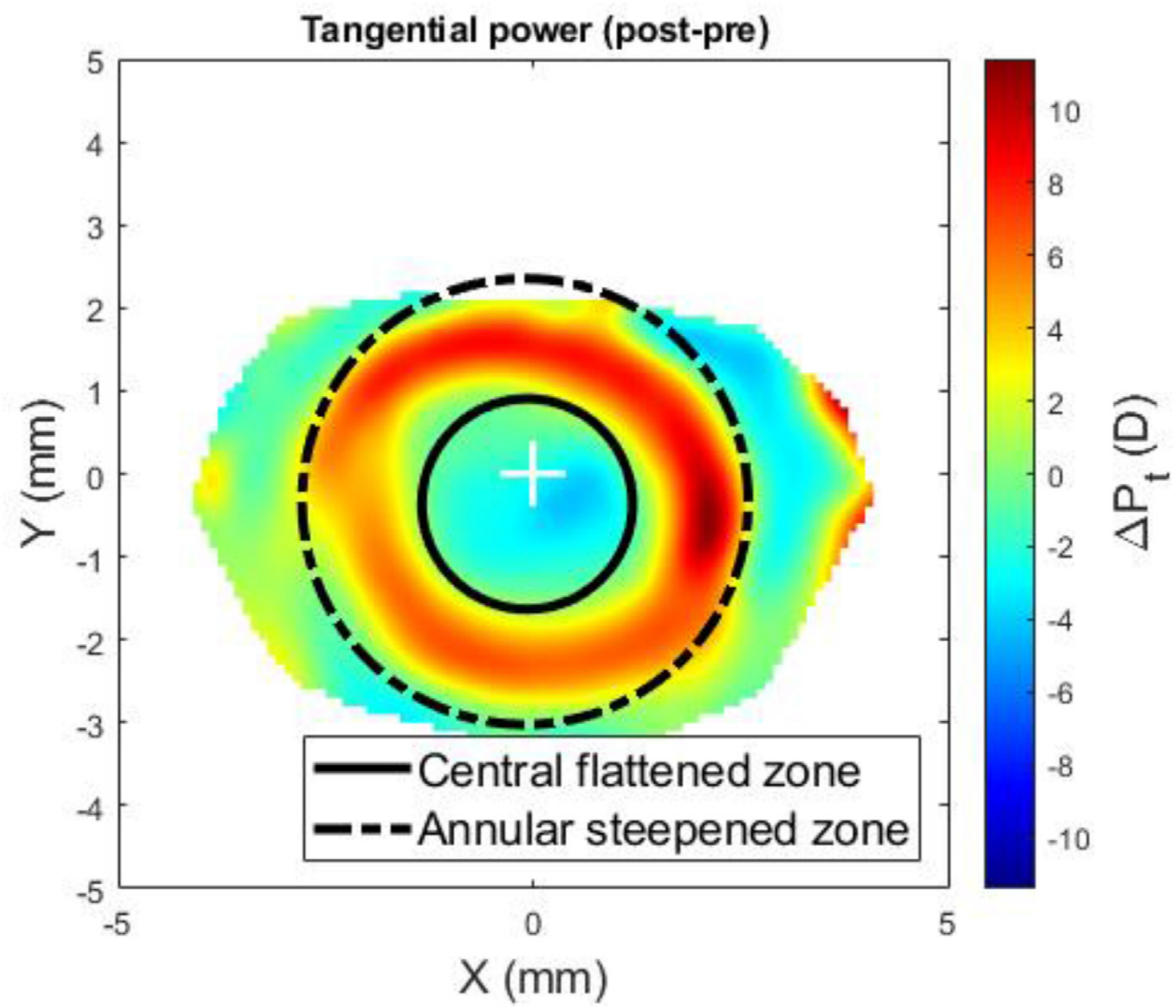
Entire outlines of the central flattened and annular steepened zones after both being fitted to circles and plotted on top of the power difference map ΔPt.

Once the power differential map of $\Delta P_{t}$ was created, the central flattened zone was automatically detected by locating the intersection between the pre and post-Ortho-K tangential refractive power at each meridian, Figure 2, then fitting the resulting scattered points to a circle using the least squared error method, Figure 3. The annular steepened zone was detected using the same method with extra filters applied to avoid picking up any noise effect. This method ensured that the automated process of power line intersection detection for the annular steepened zone ignored any points detected within the central flattened zone or that were very close to the edges of the power map to avoid any edge effect (Abass et al., 2019).

### 2.5 Smoothing

The tangential power maps were smoothed using the robust discretized smoothing spline method (Garcia, 2010). Different degrees of smoothing are applied using a positive scaling parameter S, where higher S values provide a smoother map. The method, which is based on the discrete cosine transform (DCT), works with equally spaced data in two dimensions. To ensure optimal smoothing, whereby the maximum amount of data is preserved without the disadvantage of excessive digital noise, a preliminary investigation established a value of S of 4.5 for tangential maps (see S1).

### 2.6 Statistical analysis

The statistical analysis carried out on the results of this study was performed using the Statistics and Machine Learning Toolbox of MATLAB software. The null hypothesis, at 95.0% confidence level testing, was used to investigate the inferences of the findings based on statistical evidence. The normal distribution of the samples was confirmed using the Kolmogorov-Smirnov test (Marsaglia et al., 2003) and then the two-sample t-test was applied to investigate whether there was a significance between pairs of data sets and to confirm whether the assessed findings represented an independent record. The probability value (p) is an element in the closed period 0.0 to 1.0 where values of p higher than 0.05 indicate that the null hypothesis cannot be rejected (Everitt and Skrondal, 2010). The MATLAB function 'ttest2′ was used, and the returned p-value, in addition to binary test decision for the null hypothesis. The correlation coefficient used in this study (R) is a measure of the linear dependence of two variables (Kendall and Stuart, 1973). R values below 0.4 were considered as an indication of weak correlations; R values in the range 0.4–0.6 were considered as an indication of moderate correlations; and finally, R values above 0.6 were considered as an indication of strong correlations (Evans, 1996).

## 3 Results

While averaged simulated keratometry (Sim-K) dioptric values were reduced in both flat and steep meridians as a result of Ortho-K wear, flat meridians lost 1.55 D and steep meridians lost 1.25 D, 0.3 D less than flat meridians. In terms of asphericity, flat meridians lost 0.32 while steep meridians gained 0.03 as a result of lens wear.

The pre-treatment refractions and BCVA for each Group were quite similar, with only the cylinder element for G2 showing a slight difference. Both pre and post-treatment refractions are shown in Table 2.

**Table 2:** Refraction and visual acuity data.

Groups	Pre-wear refraction				Post-wear refraction			Post-wear VA
	Sph(D) (m ± std)	Cyl (D) (m ± std)	Axis (°) (m ± std)	BCVA (m ± std)	Sph (D) (m ± std)	Cyl(D) (m ± std)	Axis (°) (m ± std)	Without corrective lenses (decimal) (m ± std)
The first group (G1)	−2.89 ± 1.45	−0.84 ± 0.49	120.90 ± 64.99	0.95 ± 0.14	−0.65 ± 0.81	−0.87 ± 0.63	109.73 ± 66.05	0.91 ± 0.16
The second group (G2)	−2.96 ± 1.30	−1.10 ± 0.62	98.33 ± 79.48	0.97 ± 0.11	−0.63 ± 0.79	−1.07 ± 0.64	86.81 ± 72.90	0.89 ± 0.17
The third group (G3)	−3.04 ± 1.44	−0.86 ± 0.46	101.39 ± 73.90	0.96 ± 0.11	−0.75 ± 0.91	−0.77 ± 0.48	100.04 ± 69.08	0.87 ± 0.18

**Figure 4:**
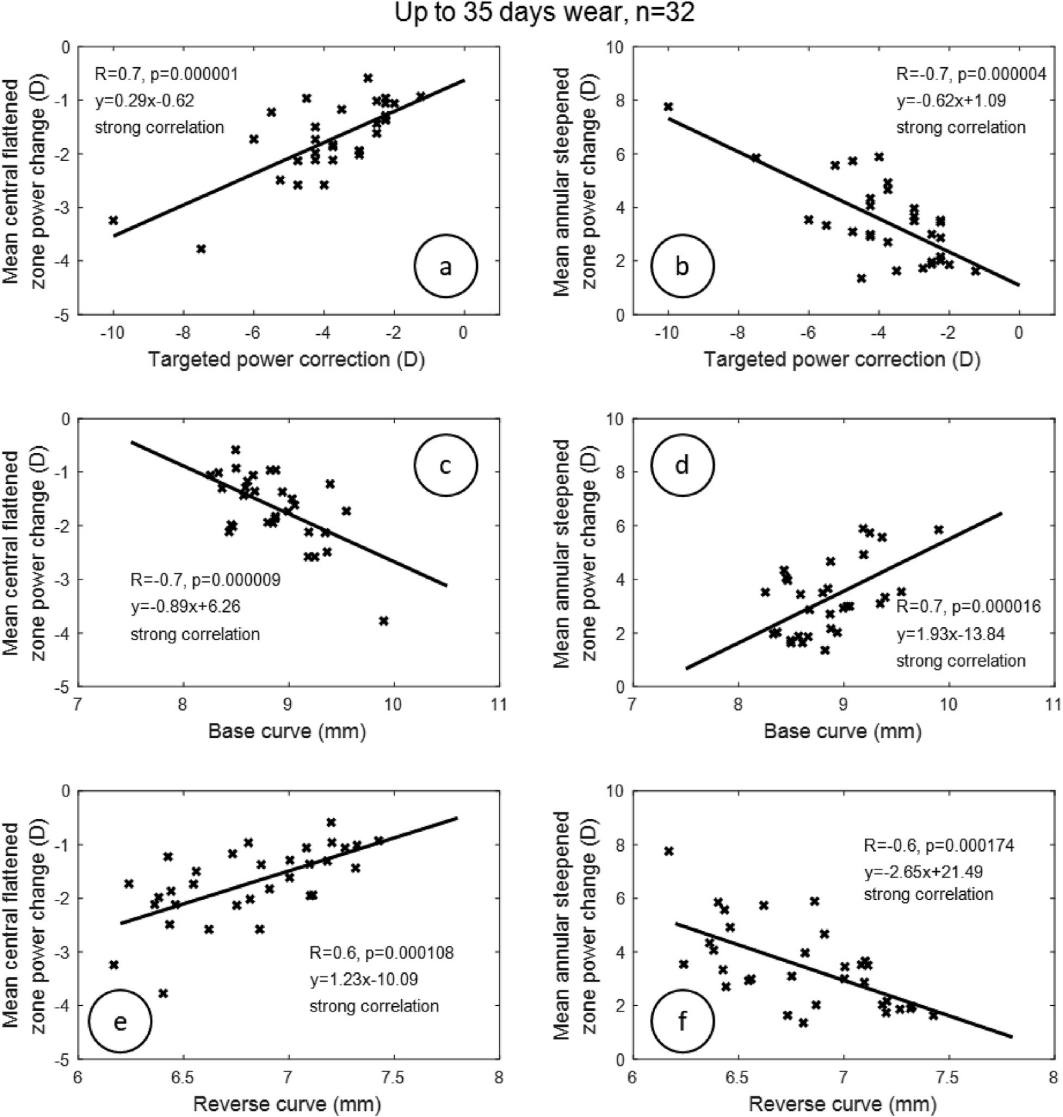
Correlation between target power correction, base curve, reverse curve and both central flattening and annular steepened power changes for the first group (G1) of participants who wore Ortho-K lenses for a time up to 35 days.

**Figure 5:**
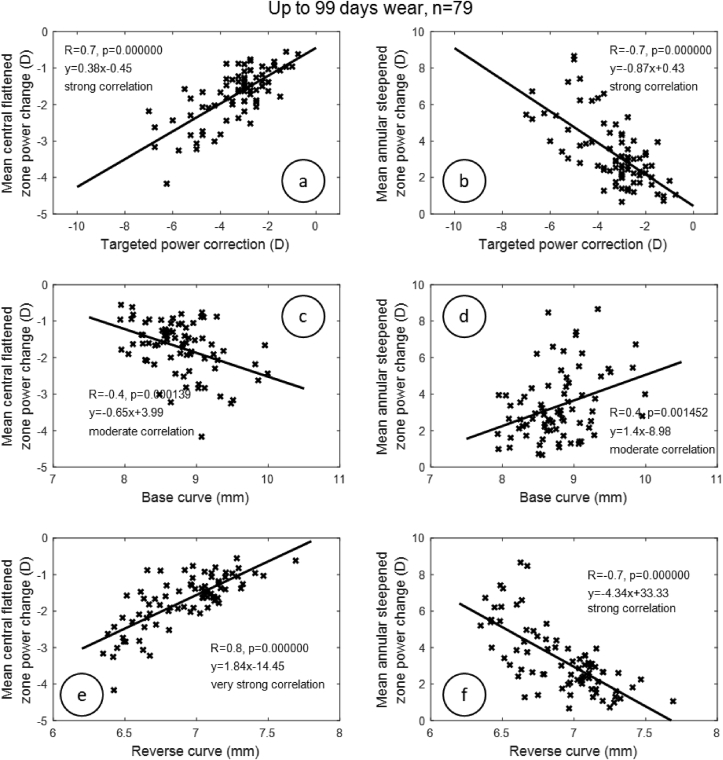
Correlation between target power correction, base curve, reverse curve and both central flattening and annular steepened power changes for the second group (G2) of participants who wore Ortho-K lenses for a time up to 99 days.

**Figure 6:**
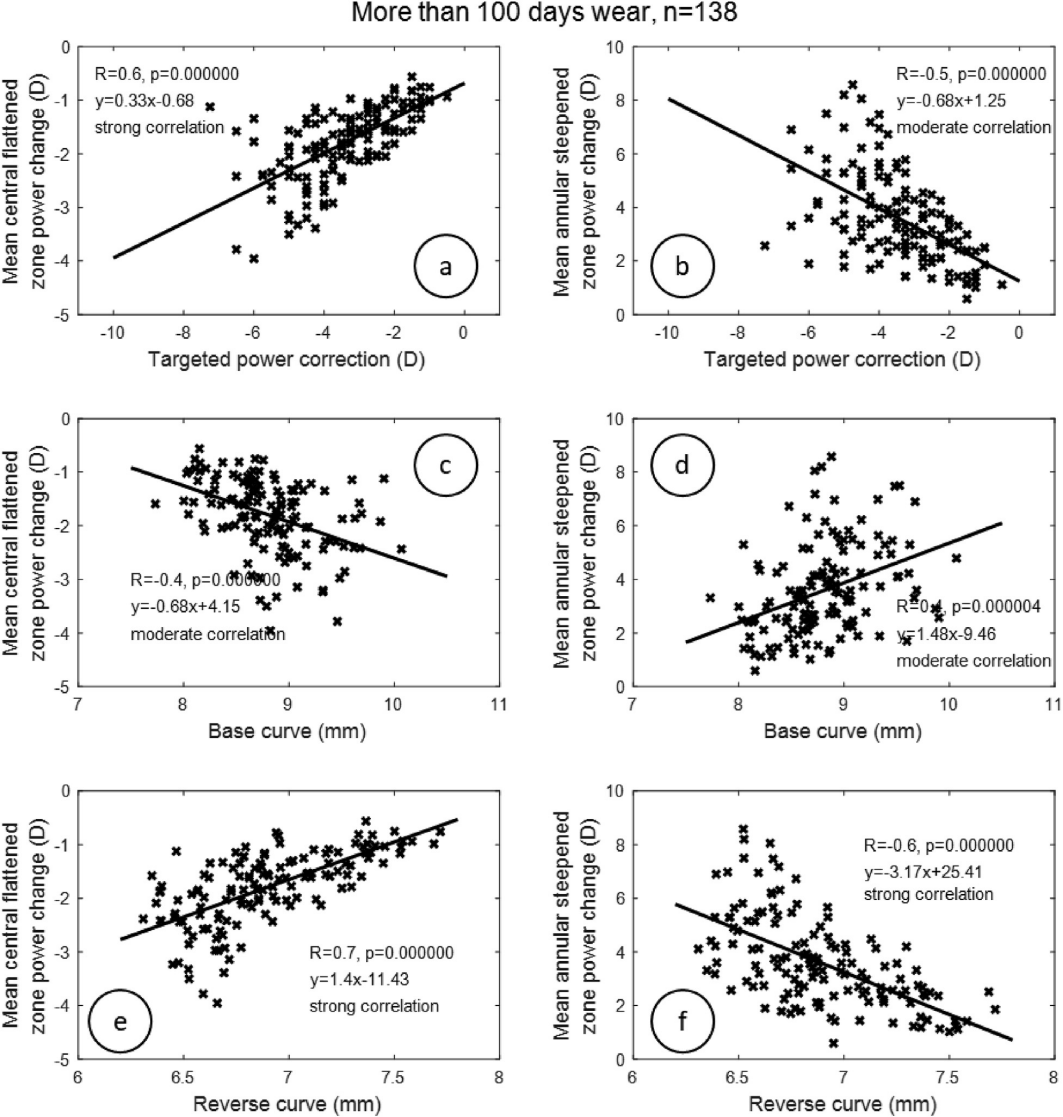
Correlation between target power correction, base curve, reverse curve and both central flattening and annular steepened power changes for the third group (G3) of participants who wore Ortho-K lenses for a time up to 500 days.

Investigation of the linear dependence between mean central flattened zone and mean annular steepened zone power changes, and then targeted power correction, base curve, and reverse curve radii, revealed a strong correlation between refractive targeted power and mean central flattened zone power change in G1 (R = 0.7, p < 0.001), G2 (R = 0.7, p < 0.001) and G3 (R = 0.6, p < 0.001); Figure 4, Figures 5 and 6. When the correlation between refractive targeted power and mean annular steepened zone power change was investigated, similar results were observed, where a strong correlation was noticed in G1 (R = 0.7, p < 0.001) and G2 (R = 0.7, p < 0.001). However, a moderate correlation was noticed in G3 (R = 0.5, p < 0.001). The radius of the base curve was strongly correlated with the mean central flattened zone power change in G1 (R = 0.7, p < 0.001) but moderately correlated with G2 (R = 0.4, p < 0.001) and G3 (R = 0.4, p < 0.001). When correlation with the mean annular steepened zone power change was considered, a strong correlation with the base curve was recorded in group G1 and a moderate correlation in G2 (R = 0.7, R = 0.4) and G3 (R = 0.4) with p < 0.001. The reverse curve was strongly correlated to the mean central flattened zone power change among all groups G1 (R = 0.6, p < 0.001), G2 (R = 0.8, p < 0.001) and G3 (R = 0.7, p < 0.001). The reverse curve was also strongly correlated with the mean annular steepened zone power change in all groups G1, G2 and G3 (R = 0.6, R = 0.7 & R = 0.6) respectively (p < 0.001). As all the groups have the same demographic characteristics, a similar age range, simulated keratometry and asphericity (as a measure of the shape), these were not considered as confounding factors for the obtained results.

**Figure 7:**
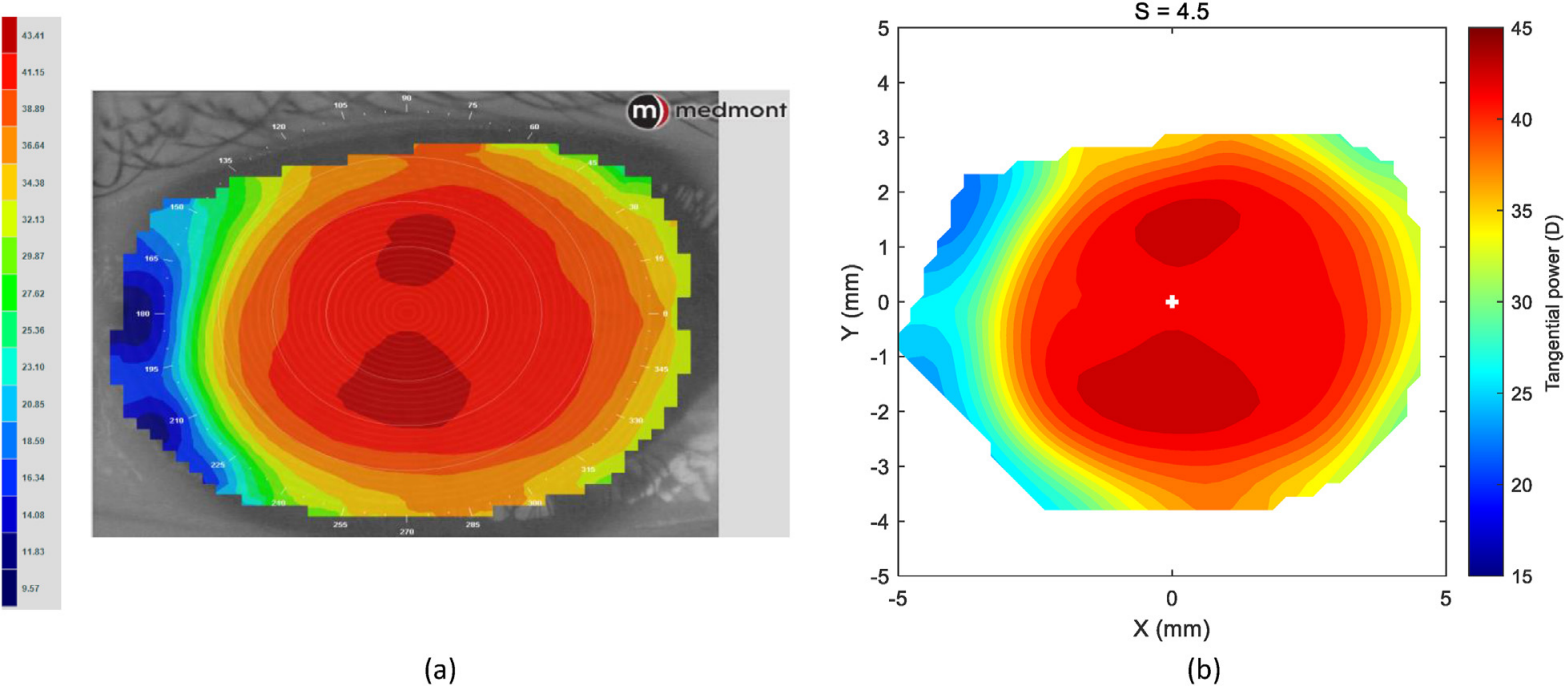
Tangential power map of left eye of a 30-year-old male subject (a) as measured by Medmont, (b) as calculated then smoothed by a custom-built MATLAB software code.

**Figure 8:**
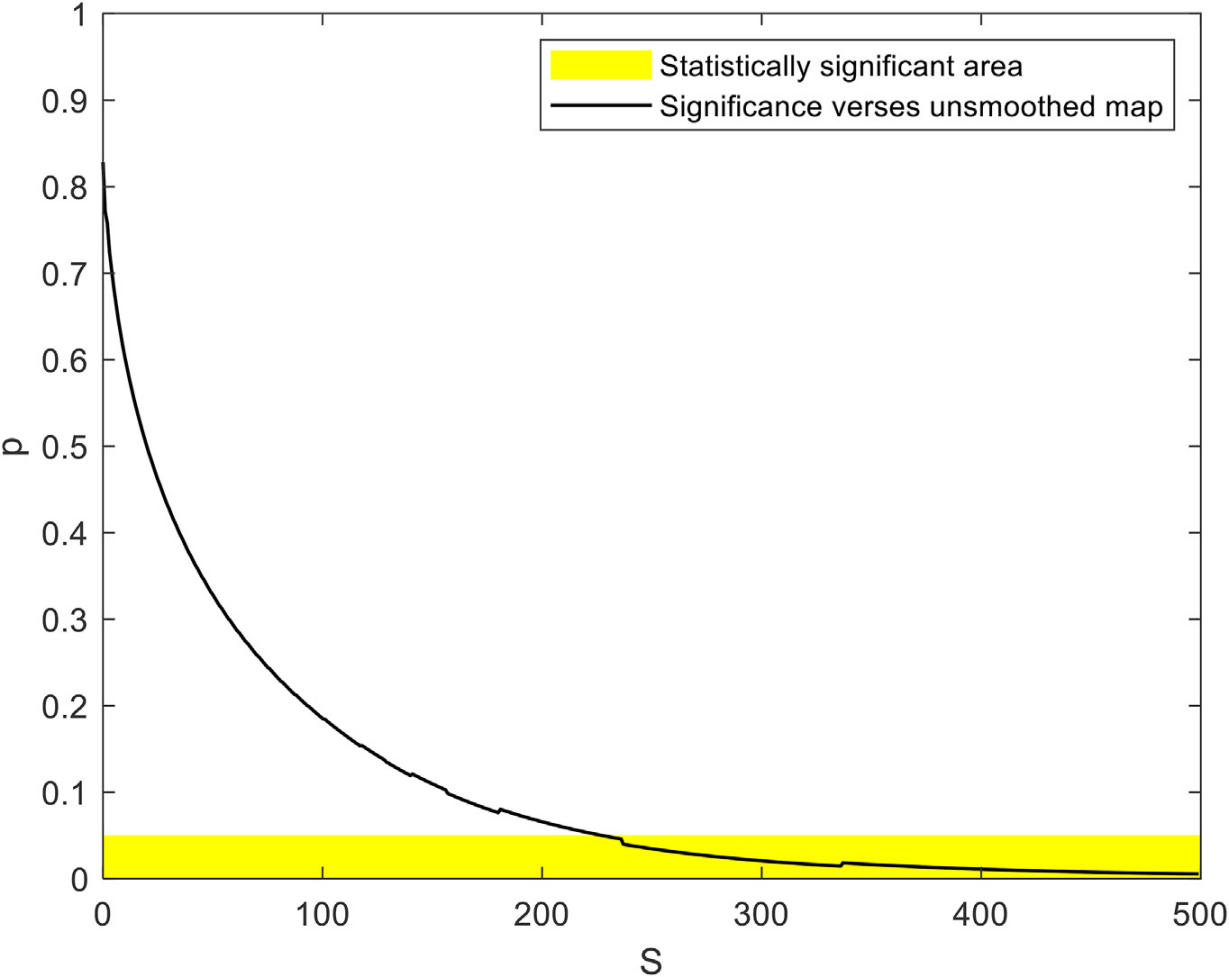
A preliminary investigation where S was changed from S = 0, which represents no smoothing, to S = 500, which represents very high smoothing and the significance (p) of difference among smoothed tangential maps and the unsmoothed map.

**Figure 9:**
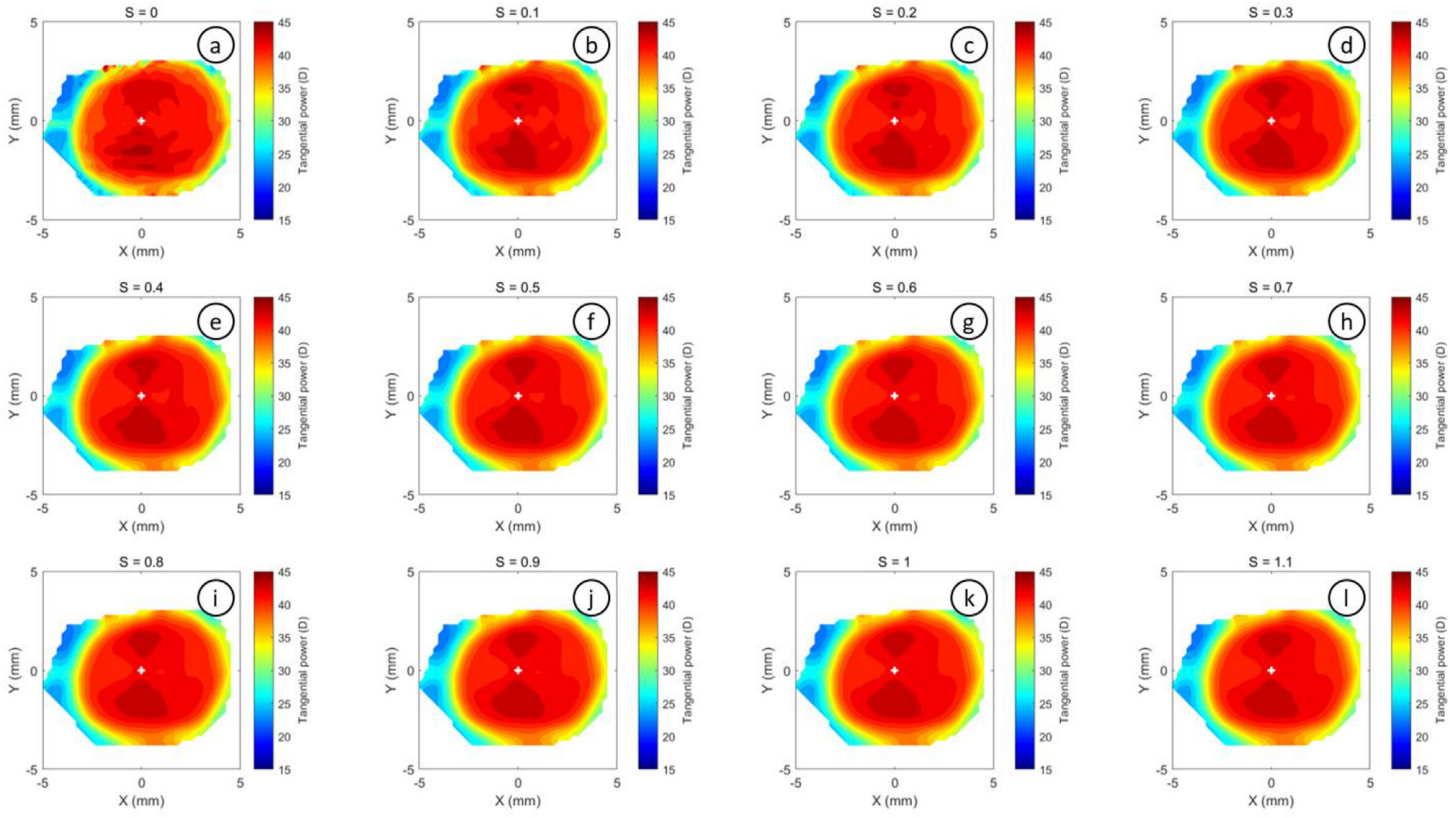
Tangential refractive power map of a 30 years male subject as calculated then smoothed by a custom-built MATLAB software code with smoothing factor S = 0 to S = 1.1.

**Figure 10:**
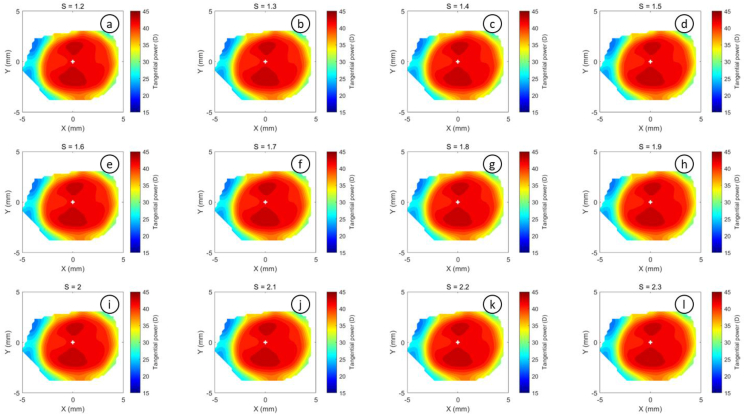
Tangential refractive power map of a 30 years male subject as calculated then smoothed by a custom-built MATLAB software code with smoothing factor S = 1.2 to S = 2.3.

**Figure 11:**
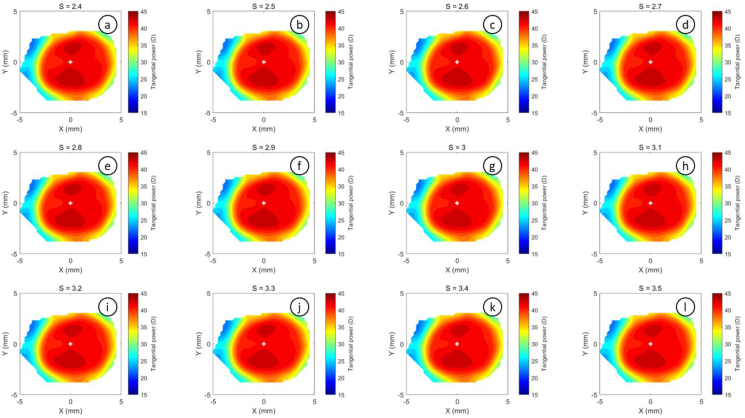
Tangential refractive power map of a 30 years male subject as calculated then smoothed by a custom-built MATLAB software code with smoothing factor S = 2.4 to S = 3.5.

**Figure 12:**
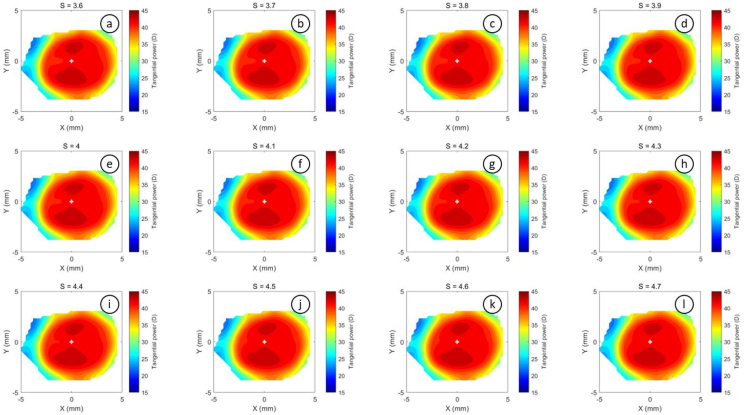
Tangential refractive power map of a 30 years male subject as calculated then smoothed by a custom-built MATLAB software code with smoothing factor S = 3.6 to S = 4.7.

**Figure 13:**
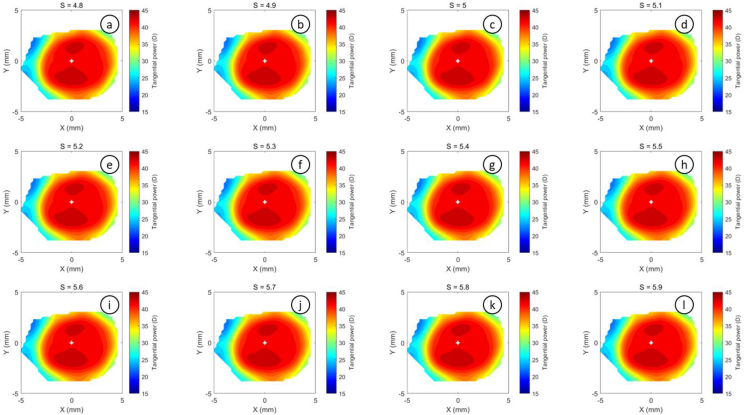
Tangential refractive power map of a 30 years male subject as calculated then smoothed by a custom-built MATLAB software code with smoothing factor S = 4.8 to S = 5.9.

**Figure 14:**
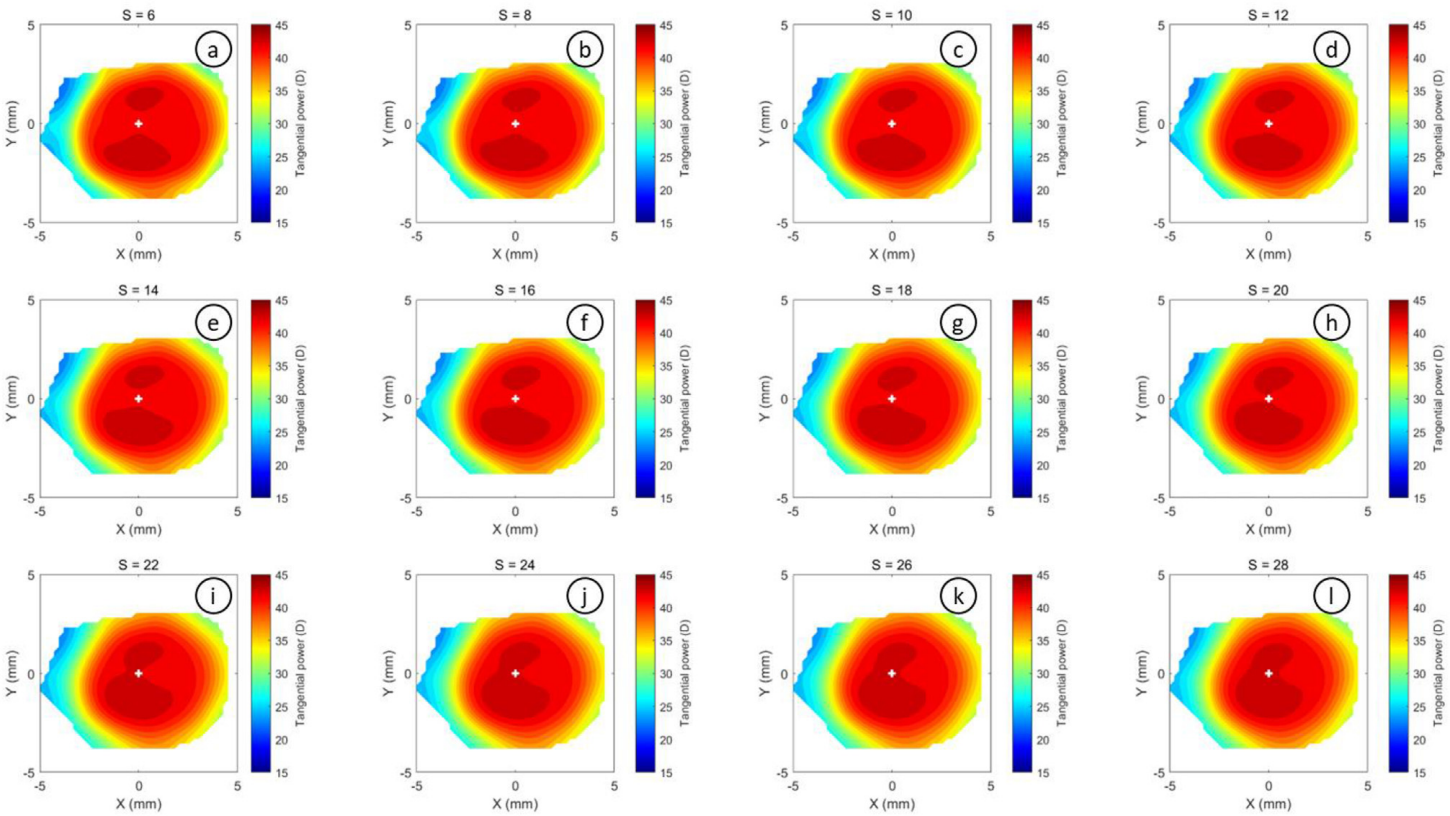
Tangential refractive power map of a 30 years male subject as calculated then smoothed by a custom-built MATLAB software code with smoothing factor S = 6 to S = 28.

**Figure 15:**
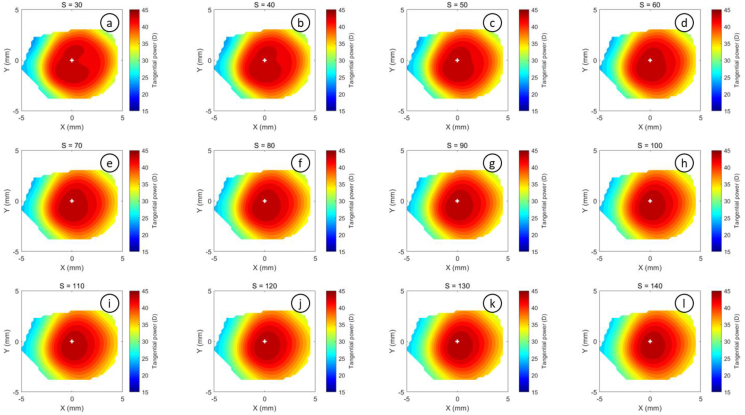
Tangential refractive power map of a 30 years male subject as calculated then smoothed by a custom-built MATLAB software code with smoothing factor S = 30 to S = 140.

The refractive power maps were created, via tangential curvatures taken from data extracted from the raw Medmont elevation data. Refractive power maps were constructed from tangential curvature data calculated from and then smoothed using varying degrees of smoothing, Figure 7 (see S1 for more details). Digital noise is always generated in the scanning process, so a preliminary investigation into the effect of smoothing the resulting refractive power maps was conducted. The aim was to reduce the noise as much as possible whilst ensuring no key information was lost in the process. This exercise showed that the second derivative created significant digital noise and as a result, tangential maps needed to be smoothed. The data suggested that the curvature map displayed by the topographer software had already been smoothed, although there is no mention of this process in the Medmont user manual. This is evident in maps with minimal smoothing where the digital noise, systematically generated by software calculations, significantly reduces the practicality of using them for diagnosis (see Figure 8, Figure 9, Figure 10, Figure 11, Figure 12, Figure 13, Figure 14, Figure 15).

## 4 Discussion

This retrospective study examines the links between corneal changes induced by the wearing of Ortho-K contact lenses as identified by topography difference maps using a custom-built software measuring methodology. The patient base was split into 3 groups, those wearing lenses for around one month, those wearing them for three months, and the remainder were long-term wearers. The study does not attempt to correlate corneal changes with myopia control, rather it examines the relationship between the contact lens parameters and the physical changes on the cornea using a software methodology that is independent of the topography machine software. In this study, the decentration of the TZ was not examined.

Each of the three groups had similar pre-wear refractions and visual acuity, with G2 having slightly higher mean astigmatism, which was reflected in higher post-treatment refraction results. This, in turn, led to a mean reduction in final visual acuity (without correction) after treatment. The G3 group represented patients who had worn the contact lenses for the longest period of time and here, the final spherical refractive error was around −0.75DS which is also reflected in a reduced final visual acuity. Group G1, who had worn contact lenses for the least period of time, showed the best post-wear refraction and final visual acuity overall, most likely due to higher compliance, as the ortho-k treatment had just been implemented. Those in the G3 group could reasonably be expected to have the lowest compliance, and this could not be controlled in a retrospective study (Lin, 2021).

Although the central corneal power change was strongly correlated to the base curve of the lens, it represented only around 50% of the target power change. This contrasts with other studies (Chan et al., 2010) that have shown that the central corneal power change represented 0.34 ± 0.57 D less than the change in refractive error. This type of difference can be affected by treatment zone diameter and, since lenses are not worn during the day, there is rebound reversal of the flattening of the central cornea over the course of the day by as much as 0.50 D to 0.75 D. However, the annular zone power change appears to be a clearer predictor of target power change for subjects who wore lenses for around 3 months, Figure 5. For this group (G2), the change in annular power is equivalent, but opposite, to target power. For groups G1 and G3, the effect is reduced after around 4.0 D target power. This might be explained by the G1 subjects not yet experiencing the full Ortho-K effect and G3 subjects, established wearers of Ortho-K contact lenses being less likely to comply exactly with the recommended treatment regimes.

The results also suggest that the tangential curvature maps produced by the Medmont topographer device software are being smoothed considerably. Users cannot take account of any such smoothing when analyzing corneal measurements, as they have no access to proprietary algorithms. This phenomenon explains the differences in corneal and refractive changes reported by researchers when investigating Ortho-K effects using different devices (Guo et al., 2022). Dedicated topography software packages smooth the output data in different ways, depending on the instrument, to improve the usability of the instrument but without explaining the methodology to the end user. Processing extracted height data independently gives a more objective indication of corneal and refractive changes and would allow studies from differing researchers to be compared directly, thus allowing a better understanding of which parameters are most important for clinical treatment such as myopia control.

The current study has a systematic error limitation due to the use of the Medmont topographer for corneal measurements. This instrument can only measure the anterior corneal surface whereas, ideally both anterior and posterior surfaces should be measured to calculate net corneal power. To compensate for this, the corneal refractive index was set to a hypothetical value of n=1.3375. Where measurements of both surfaces are possible, a corneal refractive index of 1.376 with an aqueous refractive index of 1.336 should be used, along with the central corneal thickness, to determine the net corneal power using the Gaussian optics formula (Olsen, 1986; Ho et al., 2008; Abass et al., 2018; Fathy et al., 2021).

It should also be noted that asphericity measurements provided in the current study were extracted from the Medmont E300 software (Table 1), and Placido-disk topography systems such as this have their methodical limitations (Gonzalez-Meijiome et al., 2004). They cannot measure the corneal central surface within the first mire ring, and as a result, this central region must be interpolated by the device software. Placido-disk instruments such as the Medmont E300 use imaging captured from reflected light from the tear film rather than directly measuring the surface of the cornea. Any inconsistencies in the tear film due to dry eye conditions or ocular disease can affect the accuracy of the collected data. This inconsistency of measurement becomes an essential limitation that prevents Placido-disk topographers from being used interchangeably with other devices due to law agreement (Wang et al., 2012). Placido-disk systems data are also less accurate when mapping irregular surfaces due to their inherent assumption of intensive smoothing in the radial direction (Wang et al., 2002).

## Declaration

### Author contribution statement

Lo-Yu Wu, Louise Pellegrino Gomes Esporcatte, Wen-Kai Li, Wen-Pin Lin, Marcella Q. Salomão: Conceived and designed the experiments; Performed the experiments; Analyzed and interpreted the data; Contributed reagents, materials, analysis tools or data; Wrote the paper.

Richard Wu, Lynn White, Bernardo T Lopes, Renato Ambrósio Jr, Ahmed Abass: Conceived and designed the experiments; Analyzed and interpreted the data; Contributed reagents, materials, analysis tools or data; Wrote the paper.

### Funding statement

This research did not receive any specific grant from funding agencies in the public, commercial, or not-for-profit sectors.

### Data availability statement

Data included in article/supp. material/referenced in article.

### Declaration of interest's statement

The authors declare no conflict of interest.

### Additional information

No additional information is available for this paper.

## Appendix A Supplementary data

Supplement material S1_V3.docx Supplement material S1_V3.docx
