# Supplementary material for: Investigation of the relationship between contact lens design parameters and refractive changes in Ortho-K
Source: Heliyon. 2022 Nov 19;8(11):e11699. doi: 10.1016/j.heliyon.2022.e11699 (PMC9708631; doi:10.1016/j.heliyon.2022.e11699)
Supplement: Supplement material S1_V3.docx [file mmc1.docx]

**Supplement material S1**

Noise is a signal processing term that represents undesirable alterations in measured, captured or processed data that may happen for several reasons (Tuzlukov 2018). In numerical analysis, a form of digital noise occurs when a signal is divided by a small numerical value. This is the case in Eq 2 where the tangential curvature ($R_{t}$) was numerically calculated. The changes in the raw elevation (height) $dZ$ is divided by the small radial step $dr$ as a step towards calculating $R_{t}$.

A random noise signal that has equal intensity over different frequencies is usually called white noise, but if it has been contained within a certain frequency range, it can be described as a filtered noise signal or 'coloured noise'. In our case, white noise is dominant as no filtration was considered. With no access to the smoothing algorithm that the Medmont software uses, the only available option was to use an adjustable smoothing method such as discrete cosine transform (DCT) and compare the outcome to the Medmont power map outcome. As the degree of smoothing parameter $S$, controls the level of smoothing, a preliminary investigation was done where $S$ was changed from $S=0$, which represents no smoothing, to $S=500$, which represents very high smoothing, Figure 8.

Although it was clear that statistical significance was only noticed at $S=227$ , such a high value of smoothing is sufficient to cause an over smoothing of the tangential map features. Figures from Figure 9 to Figure 15 present the tangential refractive power map of a subject's left eye smoothed to different ranges with the scaler $S$ changing from $S=0$ to $S=140$. In the current study, $S=4.5$ was selected as it gives the closest map shape to the Medmont software-generated map. Figure 10, Figure 11, Figure 12, Figure 13, and Figure 14 are covering $S$ of 1.2:2.3, 2.4:3.5, 3.6:4.7, 4.8:5.9 and 6:28 respectively.

Figure 8: A preliminary investigation where S was changed from S=0, which represents no smoothing, to S=500, which represents very high smoothing and the significance (p) of difference among smoothed tangential maps and the unsmoothed map.


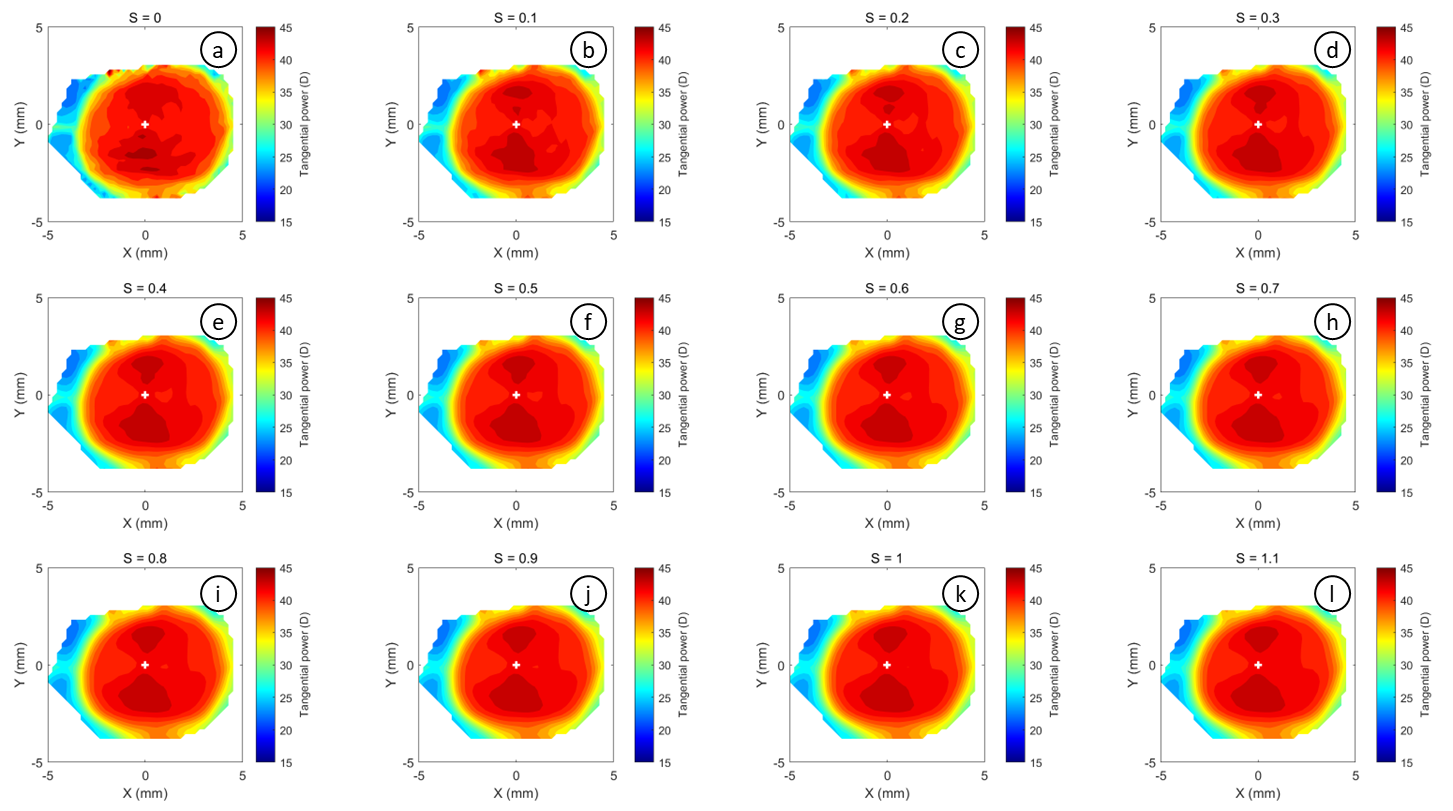


Figure 9: Tangential refractive power map of a 30-year-old male subject as calculated and then smoothed by a custom-built MATLAB software code with smoothing factor S. Subplots a, b, c, d, e, f, g, h, I, j, k & l show the effect of varying S from 0 to 1.1 in a step of 0.1 respectively.


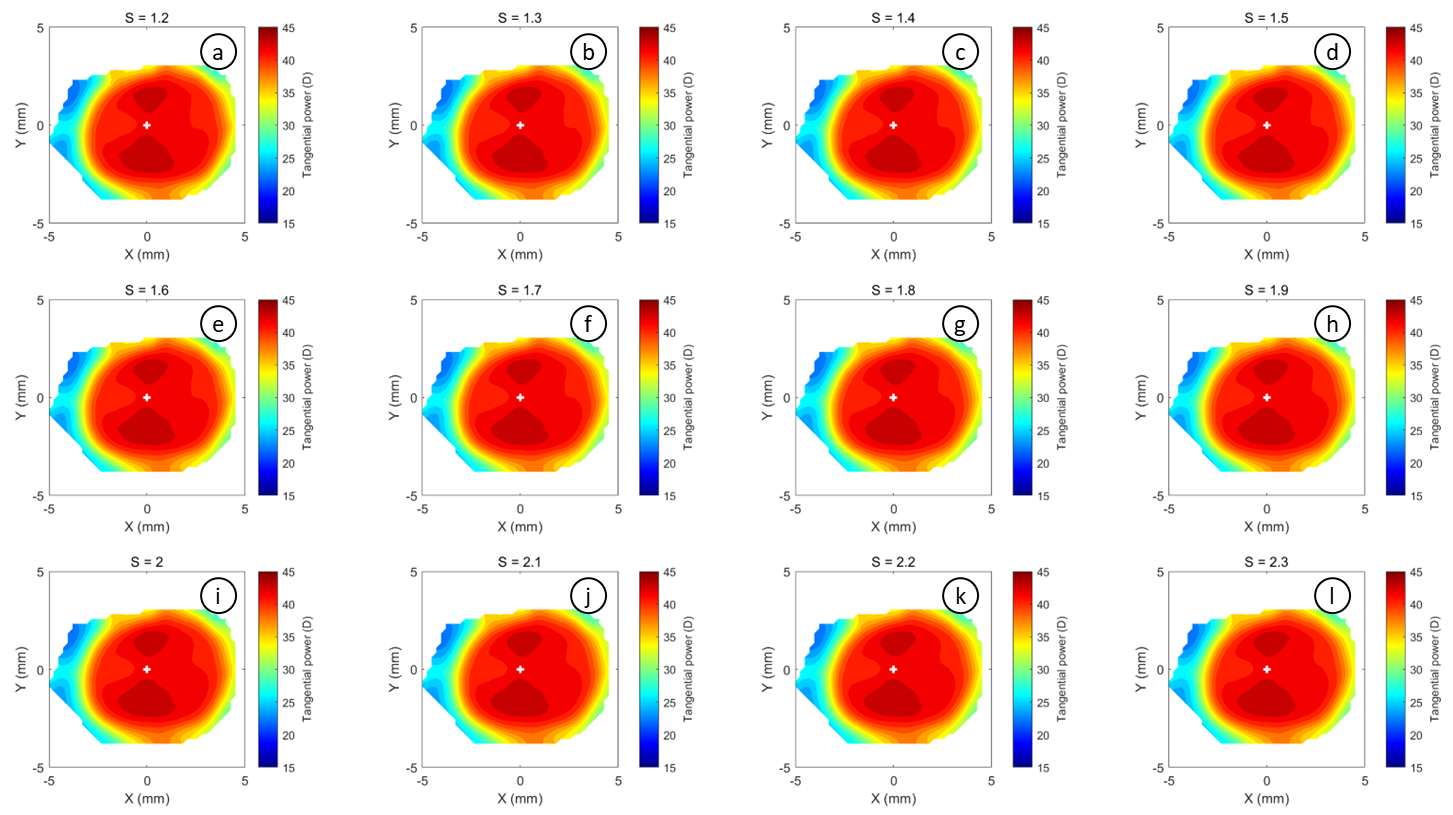


Figure 10: Tangential refractive power map of a 30-year-old male subject as calculated and then smoothed by a custom-built MATLAB software code with smoothing factor S. Subplots a, b, c, d, e, f, g, h, I, j, k & l show the effect of varying S from 1.2 to 2.3 in a step of 0.1 respectively.


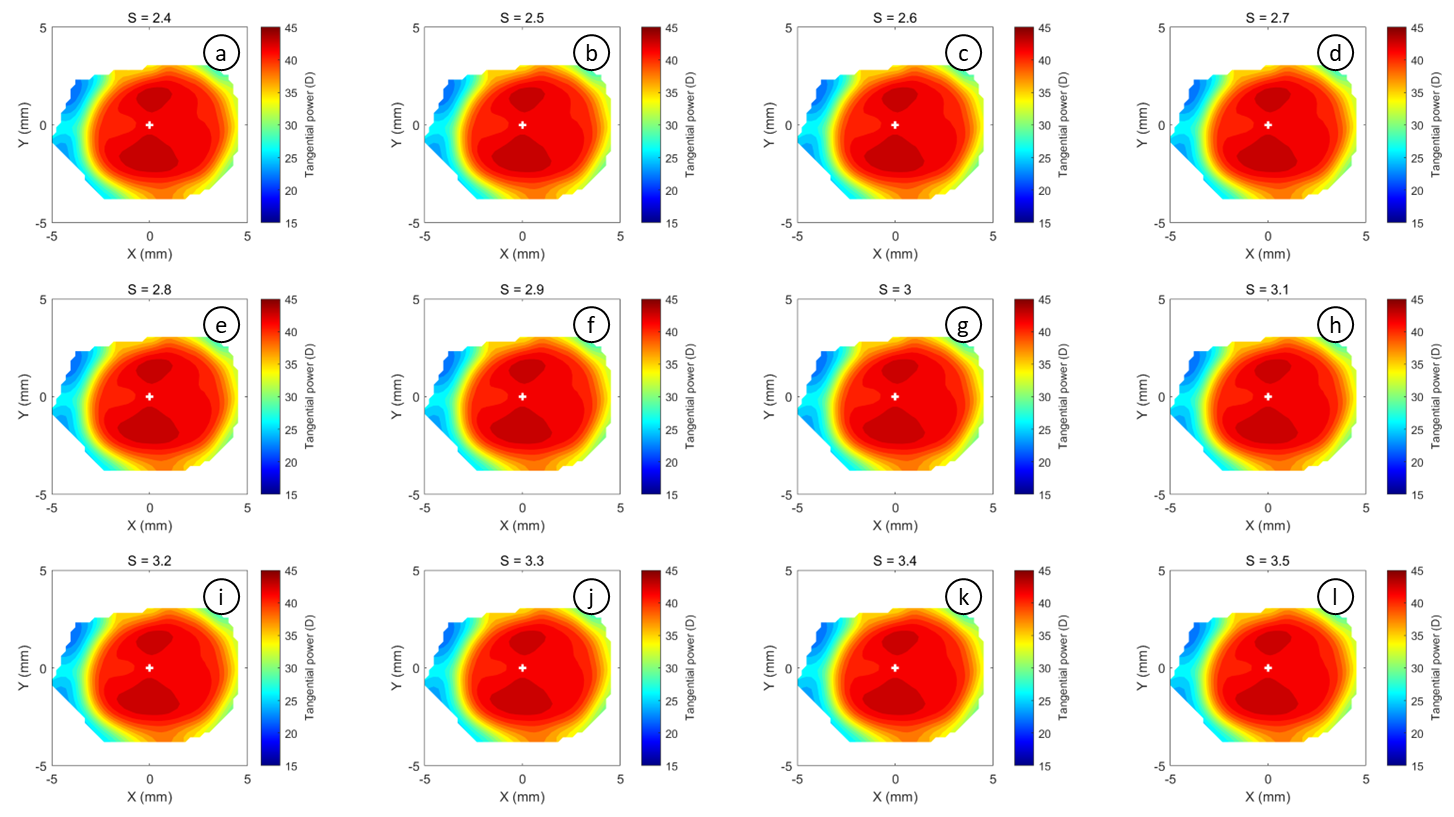


Figure 11: Tangential refractive power map of a 30-year-old male subject as calculated and then smoothed by a custom-built MATLAB software code with smoothing factor S. Subplots a, b, c, d, e, f, g, h, I, j, k & l show the effect of varying S from 2.4 to 3.5 in a step of 0.1 respectively.


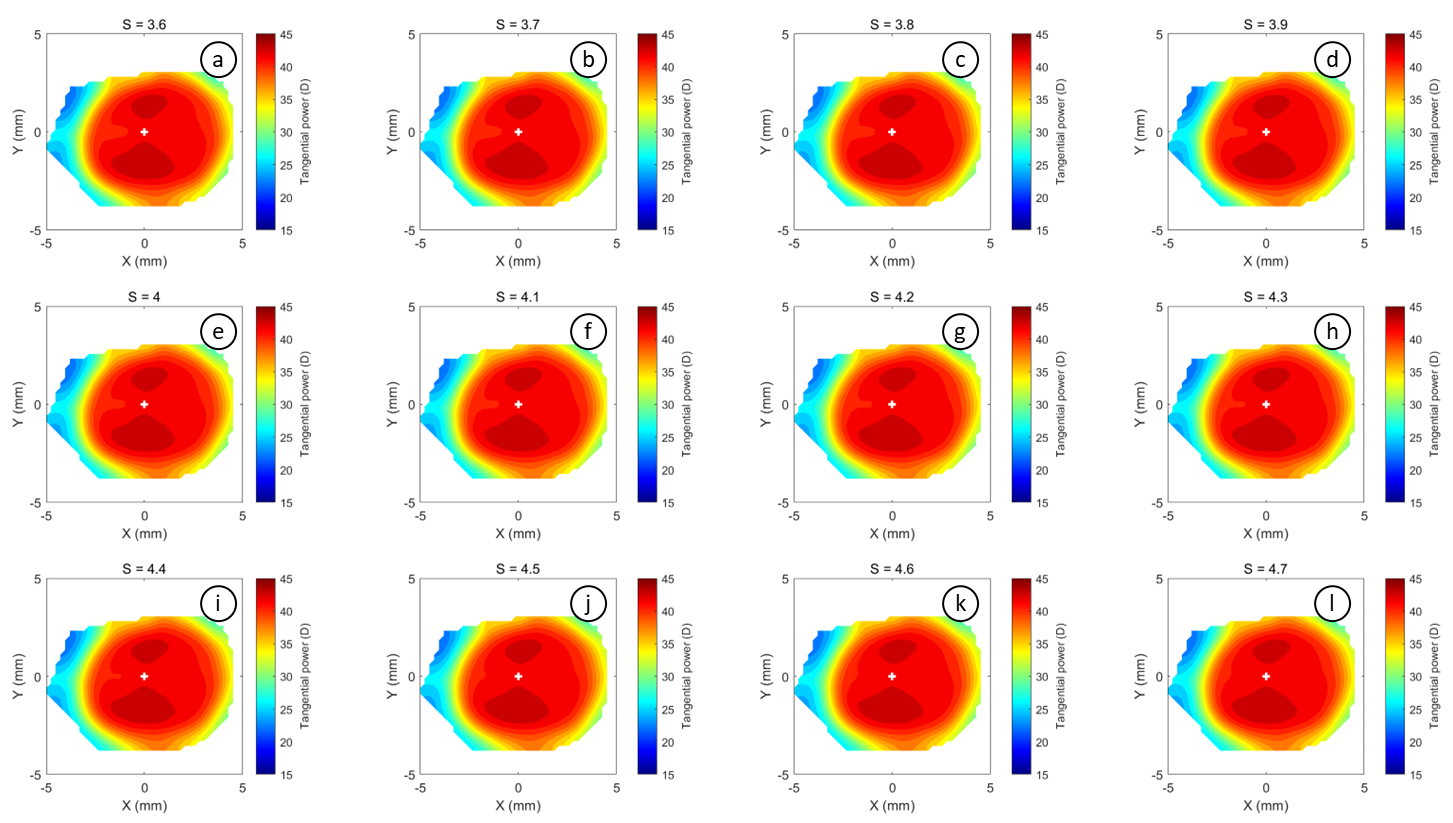


Figure 12: Tangential refractive power map of a 30-year-old male subject as calculated and then smoothed by a custom-built MATLAB software code with smoothing factor S. Subplots a, b, c, d, e, f, g, h, I, j, k & l show the effect of varying S from 3.6 to 4.7 in a step of 0.1 respectively.


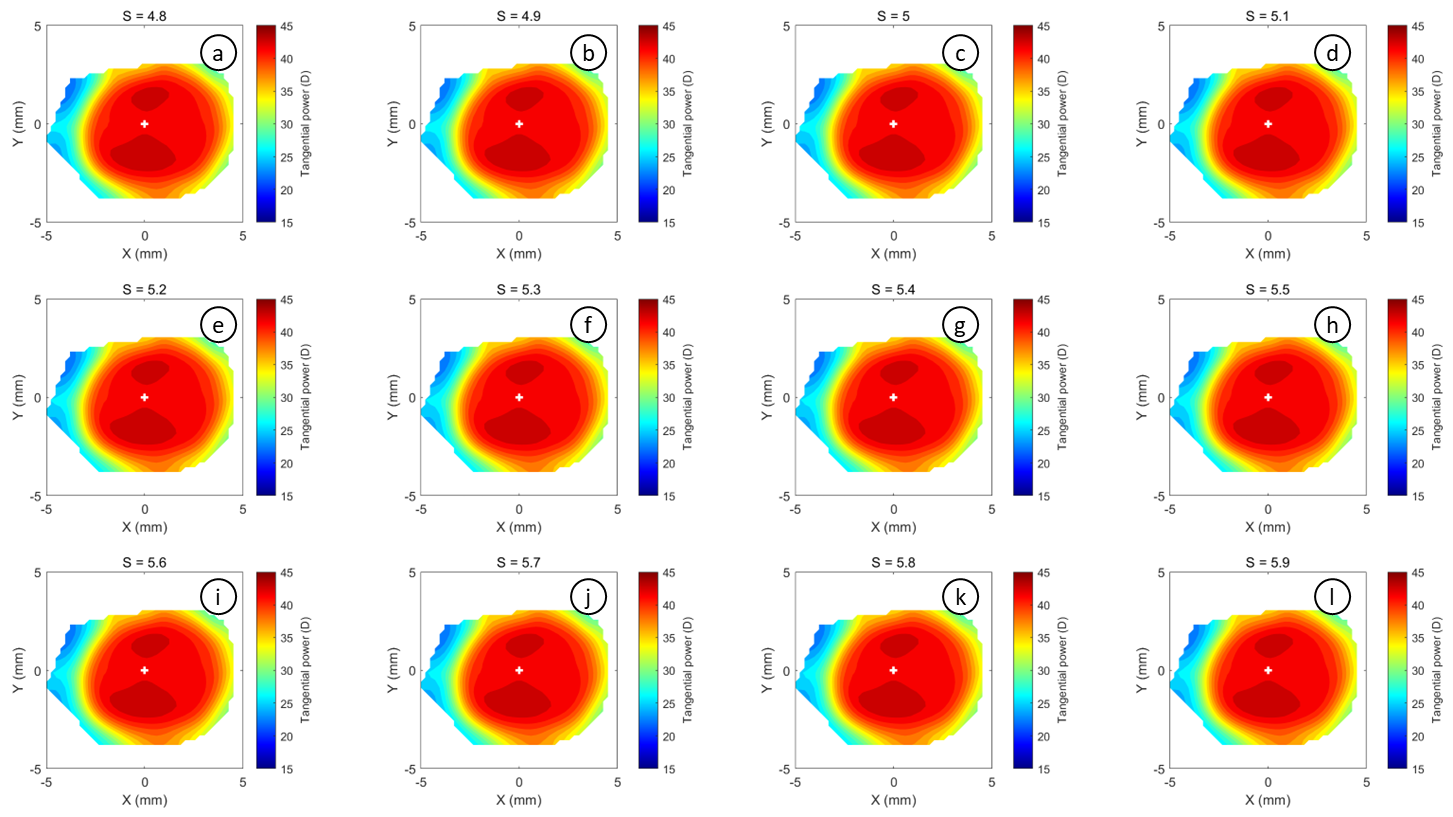


Figure 13: Tangential refractive power map of a 30-year-old male subject as calculated and then smoothed by a custom-built MATLAB software code with smoothing factor S. Subplots a, b, c, d, e, f, g, h, I, j, k & l show the effect of varying S from 4.8 to 5.9 in a step of 0.1 respectively.


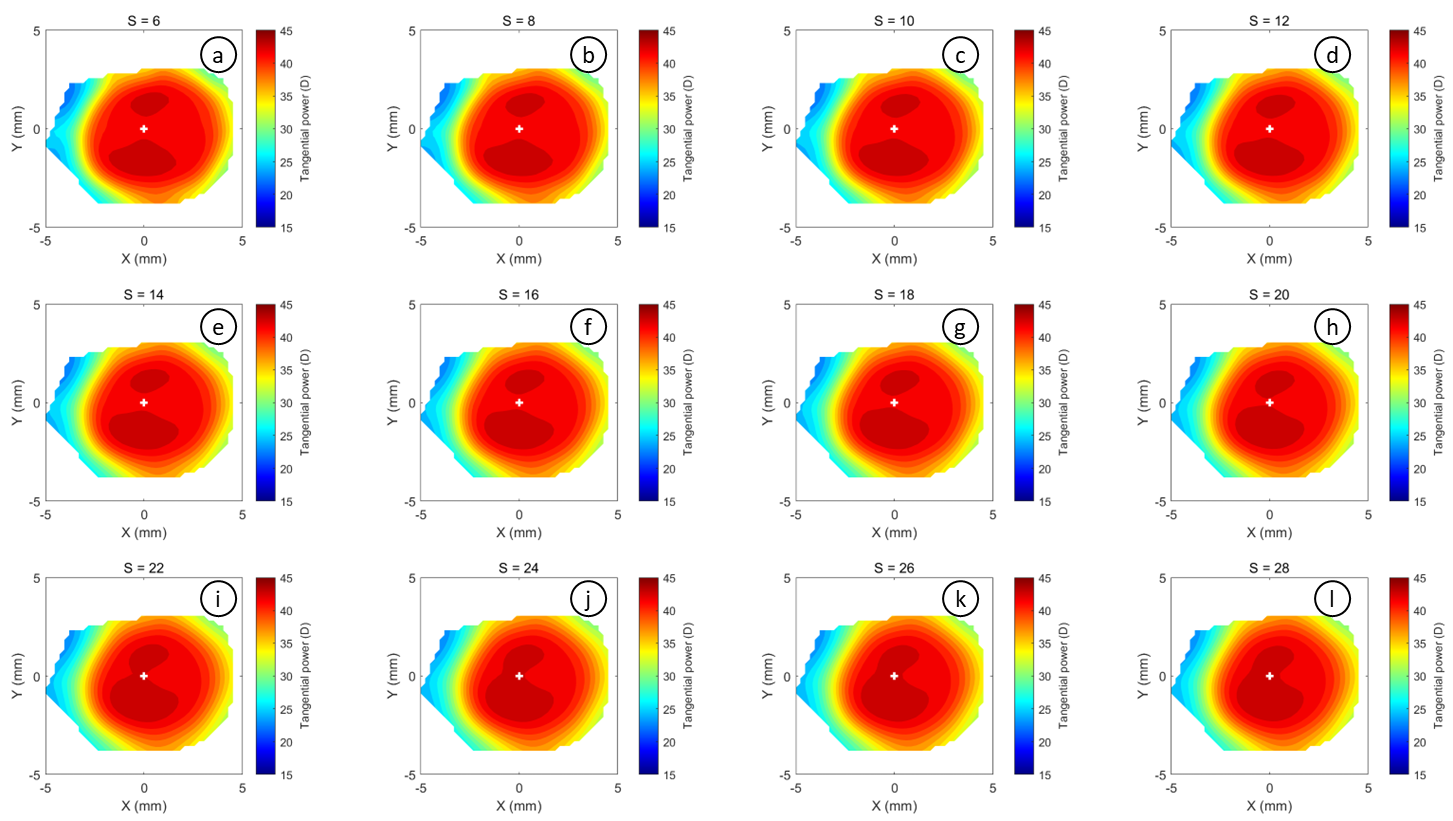


Figure 14: Tangential refractive power map of a 30-year-old male subject as calculated and then smoothed by a custom-built MATLAB software code with smoothing factor S. Subplots a, b, c, d, e, f, g, h, I, j, k & l show the effect of varying S from 6 to 28 in a step of 2 respectively.


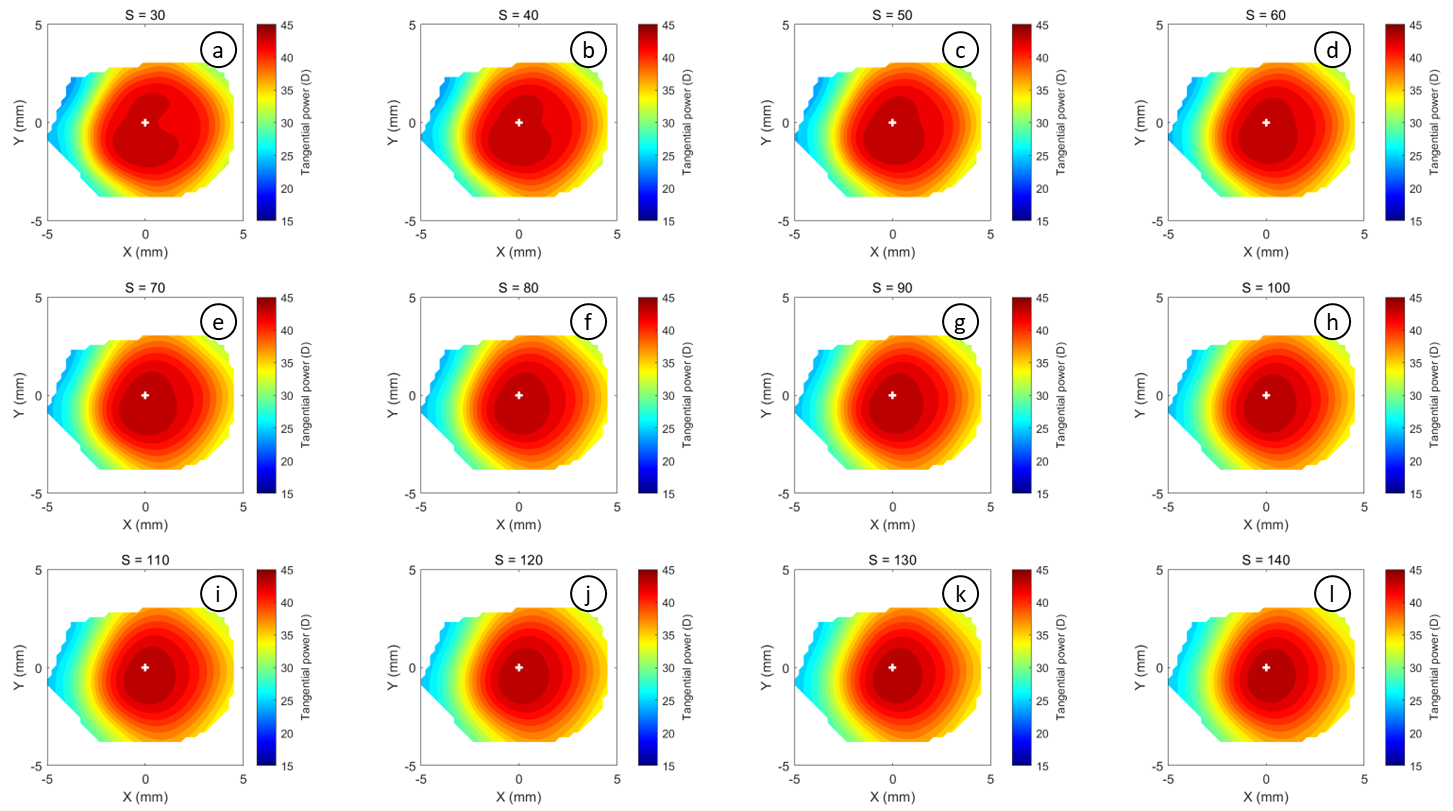


Figure 15: Tangential refractive power map of a 30-year-old male subject as calculated and then smoothed by a custom-built MATLAB software code with smoothing factor S. Subplots a, b, c, d, e, f, g, h, I, j, k & l show the effect of varying S from 30 to 140 in a step of 10 respectively.
